# Practical application of the patient data-based quality control method: the potassium example

**DOI:** 10.11613/BM.2024.010901

**Published:** 2024-02-15

**Authors:** Yan Zhang, Hua-Li Wang, Ye-Hong Xie, Da-Hai He, Chao-Qiong Zhou, Li-Rui Kong

**Affiliations:** 1 Department of Clinical Laboratory, Traditional Chinese Medicine Hospital of Pidu District, Chengdu, China; 2 Department of Clinical Laboratory, The Third Affiliated Hospital of Chengdu University of Chinese Medicine, Chengdu, China

**Keywords:** daily average value, patient-generated data, potassium, quality control

## Abstract

**Introduction:**

Internal quality control (IQC) is a core pillar of laboratory quality control strategies. Internal quality control commercial materials lack the same characteristics as patient samples and IQC contributes to the costs of laboratory testing. Patient data-based quality control (PDB-QC) may be a valuable supplement to IQC; the smaller the biological variation, the stronger the ability to detect errors. Using the potassium concentration in serum as an example study compared error detection effectiveness between PDB-QC and IQC.

**Materials and methods:**

Serum potassium concentrations were measured by using an indirect ion-selective electrode method. For the training database, 23,772 patient-generated data and 366 IQC data from April 2022 to September 2022 were used; 15,351 patient-generated data and 246 IQC data from October 2022 to January 2023 were used as the testing database. For both PDB-QC and IQC, average values and standard deviations were calculated, and z-score charts were plotted for comparison purposes.

**Results:**

Five systematic and three random errors were detected using IQC. Nine systematic errors but no random errors were detected in PDB-QC. The PDB-QC showed systematic error warnings earlier than the IQC.

**Conclusions:**

The daily average value of patient-generated data was superior to IQC in terms of the efficiency and timeliness of detecting systematic errors but inferior to IQC in detecting random errors.

## Graphical abstract

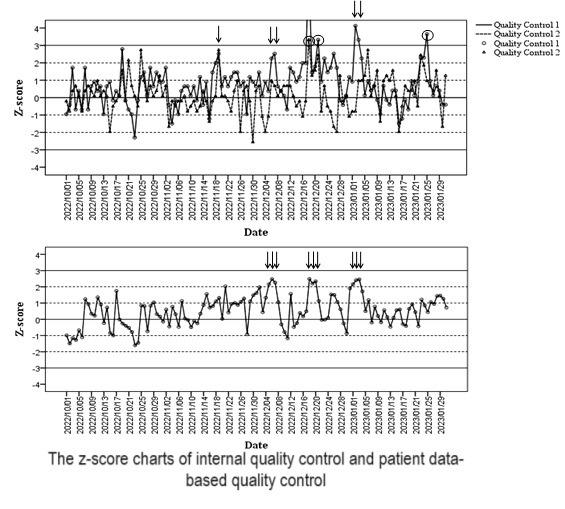

## Highlights

• The daily average calculation method might be the most convenient quality control approach to implement

• Potassium example: comparison of the effectiveness between the quality control based on the daily average value and the traditional method

• The effectiveness of the daily average value method was better for detecting systematic errors but worse for spotting random errors

## Introduction

Internal quality control (IQC) is the core pillar of laboratory quality control strategies. However, some laboratories perform IQC only once a day, which is not sufficient to detect system errors in time (*1*). Moreover, IQC materials lack the same characteristics as real patient samples and are expensive (*2*). In 1965, Hoffmann and Waid proposed the concept of average value quality control, which marked the beginning of patient data-based quality control (PDB-QC) (*3*). With research on different calculation methods and the development of software systems, a variety of algorithms have been developed (*4*). Different PDB-QC algorithms exhibit different performance characteristics when detecting different types of analytical errors (*5*). For example, the average, median, exponentially weighted average, and quartile algorithms performed well for systematic errors. However, the standard deviation and sum of outliers or “positive patient” algorithms performed well for random errors. The distribution of patient-generated data in different settings varies, as do optimal error detection algorithms (*6*, *7*). Patient data-based quality control is considered a valuable supplement to traditional IQC, and studies have shown that the smaller the biological variation, the stronger the error detection ability of patient-generated data, which is more suitable for compensating the deficiency of traditional IQC detection errors (*8*). Based on the application reports of electrolytes, serum potassium with small biological variation and high requirements for analytical performance is suitable for the exploration of PDB-QC (*6*, *8*, *9*). Therefore, on the example of quality control for measurement of potassium concentration in serum, the study compared the effectiveness between the quality control based on the daily average value and the traditional method of analyzing the commercial control samples.

## Materials and methods

### Subjects

The study period was between April 2022 and January 2023. The optimization (“training”) database consisted of 23,772 patients and 366 IQC data collected from April 2022 to September 2022. The validation (“testing”) database included 15,351 patients and 246 IQC data measured between October 2022 and January 2023. Ethical approval and informed consent were not considered necessary because no patient information entered the datasets.

### Sample collection and assays

Venous blood samples were collected from patients on the day of testing using serum vacutainers (Becton Dickinson and Company, New Jersey, USA). The samples were kept at room temperature for 30 min and centrifuged at 2650xg for 10 min to separate the serum. Serum potassium was measured using the indirect ion-selective electrode method with a Hitachi LAbOSPECT 008AS biochemical analyzer and its original reagents and calibration materials (Hitachi Hi-Tech, Tokyo, Japan). Multiqual Chemistry Controls (Bio-Rad Laboratories, California, USA) at two concentration levels (*1*, *2*) were used for IQC once a day. All calibrations as well as internal and external quality assessment (EQA) were performed according to laboratory-defined standard operating procedures. External quality assessment activities were organized by National Center for Clinical Laboratories of the National Health Commission three times a year.

### Traditional IQC method

The formula Z = (C – C)/SD was used to convert the IQC results of the testing database into Z-scores to construct a chart, where C is the daily IQC result of the testing database; C and SD are the average and standard deviation, respectively, calculated from the daily IQC results of the training database (*1*).

### Daily average value method for patient-generated data

Elimination of outliers: The boxplot method was used to optimize the data and eliminate the impact of outliers on accurate identification of errors (*10*). Data beyond the upper and lower limits were excluded from analysis. The upper and lower limits were calculated for the entire training database based on the following equations and were used to exclude outliers from both the training and testing database. Upper limit = Q3 + 1.5 × (Q3 - Q1) and lower limit = Q1 - 1.5 × (Q3 - Q1), where Q1 is the lower quartile and Q3 is the upper quartile (*10*).

Quality control chart: The formula Z = (D – D)/SD was used to convert the daily average value of the testing group into Z-scores to construct a chart, where D is the daily average value of the testing data and D and SD are the average and standard deviation of the daily average value of the training database, respectively.

### Quality control rules and data analyses

According to the Westgard-Sigma rules, 1-3S rules were rejected and judged as random errors if any z-score exceeded three, and 2-2S rules were rejected and judged as systematic errors if two z-scores exceeded two from the target values in the same direction (*1*). S denotes the standard deviation. Z-score charts were used to compare the ability and timeliness of the two methods in detecting systematic and random errors.

### Statistical analysis

For statistical analysis of the data WPS Excel 2019 (Kingsoft Office Software, Beijing, China) and SSPS23 (IBM, New York, USA) software were used. The boxplot method was used to exclude outliers. Kolmogorov-Smirnov test was used to test the normality of the data distribution. An independent-sample t-test was used to compare the differences between the two databases.

## Results

### Distribution of the patient-generated data

The serum potassium concentrations of the patients included in the analysis ranged from 2.7 to 5.0 mmol/L, the total exclusion rate of the data was 3.4%. The average number of patient-generated data points *per* day was approximately 124, and the minimum and maximum daily numbers were 34 and 186, respectively. Both the training database (P = 0.192) and the testing database (P = 0.116) were normally distributed, and no significant differences were observed (P = 0.084). The detailed results of the two databases are listed in Table 1.

**Table 1:** Distribution of patient serum potassium data

**Database**	**Total data**	**The number of data after outliers exclusion**	**Exclusion rate** **(%)**	**Average (mmol/L)**	**Standard deviation (mmol/L)**
Training	23,772	22,984	3.3	3.9	0.42
Testing	15,351	14,826	3.4	3.9	0.42

### Z-score charts of IQC and patients’ daily average values

**Figure 1:**
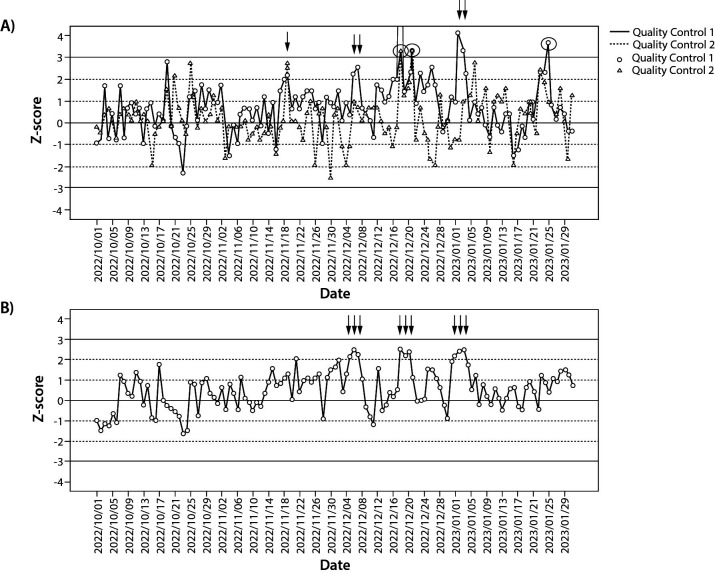
The z-score charts of IQC and PDB-QC. A: The z-score chart of IQC. B: The z-score chart of PDB-QC. Points that triggered 3S were marked with O and points that triggered 2-2S were marked with ↓. IQC - internal quality control. PDB-QC - patient data-based quality control. S - standard deviation.

The average values of potassium at IQC levels 1 and 2 in the training database were 4.0 and 7.5 mmol/L, respectively, and the standard deviations were 0.04 and 0.03 mmol/L, respectively, which were set as the target values and standard deviations of the testing database to create a Z-score chart, as shown in Figure 1 A. The average and standard deviation of the daily average value of patient-generated data in the training database were used as parameters to draw the Z-score plot for patients in the testing database, as shown in Figure 1 B.

### Comparison of the two methods for error detection

Five systematic errors and three random errors were detected using IQC. Nine systematic errors were detected in the daily average values of the patient-generated data, but no random errors were detected. Four (the 6th and 7th of December 2022, and the 2nd and 3rd of January 2023) of the five systematic errors detected by IQC were also detected by PDB-QC, and PDB-QC gave warnings one day earlier (the 5th of December 2022, and the 1st of January 2023) than IQC.

## Discussion

In this study, the errors of the analytic system were monitored by calculating the daily average value of the serum potassium concentrations in patients, which is simple and easy to implement. The efficiency and timeliness of this method in detecting systematic errors were better than those of IQC, but its ability to detect random errors was underperforming.

The serum potassium data included in the analysis were normally distributed and the daily average value of the patient-generated data was used to monitor errors in the analytic system. Due to the difference in health status among subjects, the concentration of serum potassium may fluctuate significantly, and if the coefficient of variation (CV) is too large, it will be difficult to detect small errors. Therefore, outliers in the data were eliminated and the CVs fit for this purpose. Figure 1 shows that the daily average value of serum potassium of patients provides an earlier warning than IQC in the detection of systematic errors, with better timeliness and higher detection rate. This is consistent with the daily average value of patients reported in previous studies, which has the functions of early warning and retrospective analysis (*6*, *9*). However, the ability of PDB-QC to detect random errors was not comparable to that of IQC.

The limitation of this method is that the data distribution of the test items must be analyzed to select an appropriate algorithm to ensure the reliability of the PDB-QC. According to the literature, data could not be included in the analysis when the average number of daily samples was less than 10 (*1*). The daily average sample size was about 12 times higher than 10, thus confirming the validity of the method. However, in some laboratories with small sample sizes, the difference between the daily average values of patient-generated data may be large, and their practicality requires further investigation.

In conclusion, although PDB-QC cannot replace IQC, it can be used as an effective supplement to IQC for monitoring patient results and improving laboratory quality.

## Data Availability

The data generated and analyzed are available from the corresponding author on request.
